# Clustering of longitudinal physical activity trajectories among young females with selection of associated factors

**DOI:** 10.1371/journal.pone.0268376

**Published:** 2022-05-12

**Authors:** Luoying Yang, Deborah R. Young, Tong Tong Wu

**Affiliations:** 1 University of Rochester Medical Center, Rochester, NY, United States of America; 2 Kaiser Permanente Southern California, Pasadena, CA, United States of America; Indiana University, UNITED STATES

## Abstract

We examined multi-level factors related to the longitudinal physical activity trajectories of adolescent girls to determine the important predictors for physical activity. The Trial of Activity in Adolescent Girls (TAAG) Maryland site recruited participants at age 14 (*n* = 566) and followed up with these girls at age 17 (*n* = 553) and age 23 (*n* = 442). Individual, social factors and perceived environmental factors were assessed by questionnaire; body mass index was measured at age 14 and age 17, and self-reported at age 23. Neighborhood factors were assessed by geographic information systems. The outcome, moderate-to-vigorous physical activity (MVPA) minutes in a day, was assessed from accelerometers. A mixture of linear mixed-effects models with double penalization on fixed effects and random effects was used to identify the intrinsic grouping of participants with similar physical activity trajectory patterns and the most relevant predictors within the groups simultaneously. Three clusters of participants were identified. Two hundred and forty participants were clustered as “maintainers” and had consistently low MVPA over time; 289 participants were clustered as “decreasers” who had decreasing MVPA over time; 39 participants were grouped as “increasers” and had increasing MVPA over time. Each of the three clusters has its own cluster-specific factors identified using the clustering method, indicating that each cluster has unique characteristics.

## Introduction

Physical activity is critical to promote health for both children and adults, men and women [1, 2]. However, meeting the physical activity requirements appears to be challenging for adolescent girls. National surveys consistently demonstrate that girls have lower physical activity levels than boys, and the gap increases throughout adolescence as the physical activity levels of girls drop dramatically. Both the US and global data demonstrated that less than 20% of adolescent girls met the recommended physical activity levels [3, 4], and the decline continues into adulthood [5, 6]. Adolescent girls in middle school are influenced by many factors, including school programs, supports from friends, families and community factors, such as the presence of parks, playgrounds, and other recreational facilities near home or school. In order to evaluate effective interventions and strategies to increase physical activity among adolescent girls, it is useful to identify important predictors that have a significant impact on their physical activity. Many studies have examined factors that influence physical activity participation in adolescents [7], but most used only cross-sectional designs which failed to investigate the long-term impact. In addition, physical activity is a complex behavior that is determined by factors at multiple levels [8], but only a few have included variables at the individual, social, and environmental levels [9–11], and even fewer have studied multi-level predictors of longitudinal physical activity among adolescents [12–14]. Moreover, these studies either focused on the adolescent population by including both boys and girls as a whole group [9, 10, 12] or compared the difference between boys and girls [11, 14]. One study on physical activity of middle school girls examined the differences among three racial groups: black, white and Hispanic [13]. A more in-depth and focused study was needed to examine the multi-level factors associated with the longitudinal change in physical activity of adolescent girls.

To encourage physical activity levels among adolescent girls, the National Heart, Lung, and Blood Institute initiated the Trial of Activity in Adolescent Girls (TAAG), a multi-center group-randomized trial [15]. The goal of TAAG was to determine if an intervention that provides opportunities for physical activity by linking schools to community organizations could reduce the age-related decline in moderate to vigorous physical activity (MVPA) in middle school girls, and evaluate effects of the intervention on intermediate factors at the individual and environmental (school and community) levels, as well as maintenance of the intervention effect one year after the intervention ceased. The primary endpoint for evaluating the intervention was the mean difference in intensity-weighted minutes (i.e., MET-minutes) of MVPA between girls in intervention and comparison schools. The study used accelerometry, which is considered the state of the art for physical activity assessment. Study results from the main trial indicated that following the intervention, girls in intervention schools were more physically active than girls in control schools [16].

Follow-up studies from one TAAG site was initiated by Young and colleagues [17, 18] to determine predictors of physical activity over time. The data are hierarchical and longitudinal in that individual, school and neighborhood multilevel predictors were collected over three time points at ages 14, 17, and 23. Previously reported are the results that used the first two time points of the TAAG study [18–20]. In particular, the paper of Young et al. [18] fitted cross-sectional multilevel models with fixed effects (individual, social, and neighborhood level variables) and random effects (school level) to predict MVPA. Zook and colleagues [19] divided participants into four groups based on their daily MVPA in the first two waves: (1) Active Maintainers (active at both wave 1 and wave 2); (2) Inactive Maintainers (inactive at both wave 1 and wave 2); (3) Adopters (inactive at wave 1, active at wave 2); and (4) Relapsers (active at wave 1, inactive at wave 2), where “active” was defined as MVPA greater or equal to 30 minutes each day and “inactive” was defined as MVPA less than 30 minutes each day. The paper of Grant et al. [20] examined associations among longitudinal, multilevel variables and girls’ physical activity to determine the important factors for physical activity change from age 14 to 17 by fitting a doubly regularized linear mixed-effects model. The most recent publication of Young et al. (2018) [17] was the first study analyzing the three waves of data collected in TAAG and identified a set of eight variables associated with the longitudinal trajectories of MVPA for the entire study sample using a LASSO-penalized linear mixed-effects model. A clustering procedure was conducted as the secondary analysis to further analyze this study cohort based on the MVPA trajectories and grouped the girls into three clusters with similar physical activity patterns. Their findings revealed three MVPA patterns over time: maintainers, decreasers, and increasers. It is of great interest to further understand the differences among the three MVPA patterns and the associated factors within groups, which motivated the current study. In particular, it is interesting to know the associated factors in the increaser group, which might help us understand the reasons and promote the physical activity in adolescent girls.

Therefore, we employed a novel one-step procedure to re-analyze the TAAG data to further understand the questions that haven’t been fully answered in Young et al. (2018)’s paper—what factors are associated with the different patterns of physical activity in adolescent girls. This new clustering method can overcome the limitations of two-step procedures, i.e., performing clustering and variable selection in two separate steps (detailed in the Statistical Analysis Section). We hope to gain new insight to the phenomena of decline in physical activity in the majority of girls but increase in a small subset of girls. Our new findings can potentially further inform the development of effective interventions to improve the physical activity decline that occurs during adolescence and into young adulthood. The rest of the paper is organized as follows. In the Data Description section, we describe the dataset; in the Statistical Analysis section, the analysis plan is introduced; the analysis results are presented in the Results section; and the results are explained with a discussion in the Discussion section.

## Data description

This paper is a secondary analysis of archived data from a previous study [17]. All data were fully anonymized and the IRB committee waived the requirement for informed consent. The University of Maryland Institutional Review Board approved the study when it was conducted in 2006 and 2009. For the 2015 data collection, the study was approved by the Kaiser Permanente Southern California Institutional Review Board. This review board approved the analyses that examined all of the previous data collection results. When the participants were less than 18 years of age, a parent or legal guardian provided written informed consent and the participant provided written assent. When the participant was at least 18 years of age, she provided written consent. Written consent was obtained from all participants.

TAAG enrolled 730 eighth grade girls (aged 14 years) at the Maryland Field Center in the Baltimore, MD/Washington, DC area in spring 2006. Of these, 589 (81%) were re-recruited and measured in 2009 during their 11th grade year (aged 17 years) [21]. In 2015, 460 (78%) participants (aged 23 years) were recruited and measured again [22]. Other TAAG sites did not participate in follow-up assessments. Young et al. (2018) used a socioecological model to identify potential study variables associated with MVPA [15]. More information about the 31 selected variables is summarized in the Supplementary Table, as indicated by Young et al. (2018).There were 1561 records from 568 participants (where 428 participants have three records, 137 participants have two records and three participants have only one record) with no missing values for all these variables included for modeling.

### MVPA trajectories

Daily minutes of MVPA were measured objectively with Actigraph accelerometers (MTI model 7164). The accelerometers were programmed to collect data in 30-second intervals. Girls wore the monitors for seven consecutive days on the right hip at all times except while sleeping, bathing, or swimming. Acceleration counts per 30 seconds that were above the threshold of 1,500 counts was used to determine MVPA. The participant would be asked to re-wear the accelerometer if inadequate wear was noted (<4 days, 10 hours/day). After being collected, raw data were stored in 30-second intervals. The count threshold of 3000/minutes was used to determine daily MVPA [23].

### Independent variables

There are three types of independent variables: individual-level variables, social-level variables and neighborhood-level variables.

Individual-level variables consist of demographic and personal health behavior factors. Demographics included race/ethnicity, mother and father’s education level. Cohort members self-identified as white, black/African-American, Hispanic/Latina, Asian/Pacific Islander, American Indian/Alaska Native, or other; Participants also reported mother’s and father’s education level separately which were used as the SES proxy; Cigarette-smoking behavior was evaluated using five questions adapted from the 2003 Youth Risk Behavior Survey. Personal health behavior factors included Body Mass Index (BMI), self-management strategies [24], physical activity self-efficacy [25], physical activity enjoyment [26], perceived barriers to physical activity [27], physical activity outcome-expectancy [25], and depressive symptoms [28]. BMI was calculated from measured weight and height at ages 14 and 17 and obtained from self-report at age 23, and other factors were measured using surveys.

Social variables describe social support sources for physical activity, i.e. how participants gain support for physical activity. They were assessed using three scales [29]: providing social support, friend support and family support. The three scales were measured using surveys with items like “During a typical week, how often do your friends do physical activities or play sports with you?”

Neighborhood-level variables consist of perceived neighborhood environmental factors and geographic neighborhood factors. Perceived neighborhood environmental factors were participants’ evaluations of their living environment. They were assessed using surveys. Geographic neighborhood factors were objectively measured using geographic information systems technology and spatial analysis, which provided information such as the number of parks and schools near the neighborhoods.

### Statistical analysis

#### Previous findings

The paper of Young et al. (2018) analyzed the data in two steps to understand the underlying factors with the overall trend of MVPA among adolescent girls. In the primary analysis, they used a linear mixed-effects model with LASSO penalty using the entire sample and identified eight variables related to the overall trend of physical activities over time; in the secondary analysis, a clustering method was used to group participants with similar MVPA patterns and the demographic factors at age 23 were compared across clusters. Although the goals in Young et al. differed from ours, it is still reasonable to compare the approaches in general. The two-step procedure has several limitations. The major limitation is that the assumptions of each step differ. The clustering step assumes the data were drawn from a mixture of subgroups that differ solely based on their MVPA trajectories, and the variable selection step assumes that the data was drawn from a homogeneous population. Second, because only trajectories are used in the clustering step, the relationship between MVPA and covariates is not considered. Third, it requires all trajectories to have the same length, thus participants with missing outcome were excluded from the analysis. To overcome the limitations, we proposed a one-step procedure that performs clustering and variable selection simultaneously. The one-step procedure assumes the data were drawn from a mixture of subgroups and in each subgroup the factors have group-specific associations with MVPA. As a result, it can identify subgroups based on both the MVPA trajectories and the relationship between the variables and MVPA for clustering and recognize the group-specific association between the variables and MVPA. In addition, the one-step procedure was also able to include more participants with missing outcome in the analysis.

#### One-step procedure of clustering with variable selection

A novel one-step procedure [33] was used to perform clustering and variable selection simultaneously via a mixture of linear mixed-effects models with shrinkage penalties on both fixed effects and random effects.

The problem of clustering lies in using response vectors ***y***_*i*_, fixed effects ***X***_*i*_, and random effects ***Z***_*i*_ for classification of subjects into one of the *G* groups where *G* is the total number of groups. *G* is usually unknown, and can be determined by the Bayesian information criterion (BIC). One natural approach is to assume that (***y***_*i*_, ***X***_*i*_, ***Z***_*i*_) follows a mixture of Gaussian distributions with *G* groups, and we can interpret each mixture component as a cluster. Let *π*_*g*_ be the mixing probability that indicates the probability of subject *i* being in cluster *g*, and $\sum_{g=1}^{G}\pi_{g}=1$. Then, the Gaussian mixture model and the marginal likelihood function of ***y***_*i*_ given ***X***_*i*_ and ***Z***_*i*_ has the form

$$\begin{array}{c} l\left(y_{i}|X_{i},Z_{i}\right)=\sum_{g=1}^{G}\pi_{g}\phi \left(y_{i}|\mu_{g}\left(X_{i}\right),\Sigma_{g}\left(Z_{i}\right)\right) \end{array}$$ (1)

where *ϕ* is the multivariate Gaussian likelihood function with mean ***μ***_*g*_ that includes the fixed effects ***X***_*i*_ and covariance **Σ**_*g*_ that includes the random effects ***Z***_*i*_. The forms of ***μ***_*g*_ and **Σ**_*g*_ are determined by the model within the separate cluster *g*.

Within each cluster *g*, a linear mixed-effects model was used to model the relationship between ***y***_*i*_ and ***X***_*i*_, ***Z***_*i*_ for subject *i*.

$$\begin{array}{c} y_{i}=X_{i}\beta_{g}+Z_{i}b_{ig}+\epsilon_{ig}, \end{array}$$ (2)

where ***β***_*g*_ is the fixed effects coefficients in cluster *g*, and ***b***_*ig*_ is the subject-specific random effects coefficient that follows a normal distribution $b_{ig}∼N\left(0,\sigma_{g}^{2}D_{g}\right)$, and ***ϵ***_*ig*_ is the random error that follows $N(0,\sigma_{g}^{2}I)$. Thus, the Gaussian distribution ***y***_*i*_ follows is

$$\begin{array}{c} y_{i}∼N\left(X_{i}\beta_{g},\sigma_{g}^{2}\left(Z_{i}D_{g}Z_{i}+I\right)\right). \end{array}$$

In our analysis, all 31 factors listed in the Data Description were included as fixed effects candidates for selection that account for the mean response over time. We assume random intercepts for each subject, i.e., ***Z***_*i*_ = **1**, which account for the individual difference in response over time. Including all of these variables would result in a large model that is difficult to interpret and can lead to instability in estimating parameters, thus the model adds a penalty function on the fixed effects regression coefficients to reduce the model size. The penalty function acts as a constrain while maximizing the log-likelihood to estimate ***β***_*g*_,

$$\begin{array}{c} Q_{n}\left(\Theta_{g}\right)=\text{log}\;\phi \left(y_{i}|X_{i}\beta_{g},\sigma_{g}^{2}\left(Z_{i}D_{g}Z_{i}+I\right)\right)-P_{\lambda_{1}}\left(\beta_{g}\right) \end{array}$$ (3)

where **Θ**_*g*_ is mixture-specific regression parameters of interest $\Theta_{g}=\left(\beta_{g},D_{g},\sigma_{g}^{2}\right)$, $P_{\lambda_{1}}$ is the penalty function placed on fixed effects. The penalty function shrinks the regression coefficients of the irrelevant variables to zero. As a result, only important variables can be included in the model. The number of variables included in each cluster depends on the tuning parameter λ_1_. The optimal value of λ_1_ can be determined by BIC to ensure the balance between adequate fitting and parsimony. After **Θ**_*g*_ has been updated, each subject is assigned a cluster membership *w*_*ig*_ based on its likelihood in each cluster, and *π*_*g*_ can be updated accordingly:

$$\begin{array}{c} w_{ig}=\frac{\pi_{g}\phi_{g}\left(y_{i}|X_{i},\Theta_{g}\right)}{\sum_{l=1}^{G}\pi_{l}\phi_{l}\left(y_{i}|X_{i},\Theta_{l}\right)},\;\;\pi_{g}=\frac{\sum_{i=1}^{n}w_{ig}}{n}; \end{array}$$ (4)

Once cluster membership for each subject has been updated, **Θ**_*g*_ can updated again to reflect the change in clusters. The optimal estimates can be obtained by alternating between updating **Θ**_*g*_ and *w*_*ig*_, *π*_*g*_ until the log-likelihood starts to decrease or there is little change in the estimates.

The model fitting process is summarized as: (1) standardize the data such that each variable has mean 0 and standard deviation 1; (2) start with a 2-cluster model, apply a grid of tuning parameters associated with the penalty function to the model which controls the shrinkage strength; (3) determine the optimal model that gives the minimum BIC; (4) repeat 2 and 3 for 3-cluster model, 4-cluster model and so on, obtain a optimal model for each cluster number; (5) determine the optimal model among the optimal models from each cluster number. This model gives the best cluster number and the best balance of fit and parsimony. The R packge *cluster-splmm* [30], which performs clustering of high-dimensional longitudinal data based on simultaneously penalized LMM, is available at GitHub with a simulated data example (https://github.com/lyang19/cluster-splmm). The modeling results are presented in the next section.

## Results

### Overall characteristics of the sample

The individual variables comparison is summarized in Table 1. Most individual factors and social factors changed over time. The percentage of smokers and BMI increased significantly over time, while MVPA decreased significantly over time. Self-management strategies and outcome expectancies for physical activity improved over time, while perceived barriers for physical activity, provided social support, family support and friend support declined significantly. For neighborhood factors, many participants changed residential location at age 23. Significant declines in places to walk near home, seeing kids playing in the neighborhood, interesting things to look at and having neighborhood streets that were well lit were noted, while there were increases in traffic and crime in the neighborhood. There were also increases in trails in neighborhood and the number of parks within 1 mile of their home, while the distances to the nearest park and the nearest school shortened.

**Table 1:** Demographic, behavioral, psychosocial and neighborhood characteristics at ages 14, 17, and 23 years.

	Age 14	Age 17	Age 23	*p*
	*N* = 566	*N* = 553	*N* = 442	
n (%)				
Race/ethnicity				
Black	120 (21.2)	117 (21.2)	110 (24.9)	
Other	104 (18.4)	102 (18.4)	78 (17.6)	
Hispanic	76 (13.4)	73 (13.2)	44 (10.0)	
White	266 (47.0)	261 (47.2)	210 (47.5)	
Mother’s education				
not finish high school/did not know	83 (14.7)	82 (14.8)	58 (13.1)	
High school	191 (33.7)	187 (33.8)	164 (37.1)	
College or more	292 (51.6)	284 (51.4)	220 (49.8)	
Father’s education				
Did not finish high school/did not know	115 (20.3)	116 (21.0)	85 (19.2)	
High school	208 (36.7)	199 (36.0)	170 (38.5)	
College or more	243 (43.0)	238 (43.0)	187 (42.3)	
Smoker				<0.0001
Yes	12 (2.1)	37 (6.7)	36 (8.1)	
No	554 (97.9)	516 (93.3)	406 (91.9)	
Mean (SD)				
Places to walk near home	3.63 (1.37)	3.37 (1.41)	2.97 (1.51)	<0.0001
Sidewalks in neighborhood	3.86 (1.52)	3.85 (1.47)	3.97 (1.39)	
Bike/walking trails in neighborhood	2.93 (1.59)	3.03 (1.55)	3.17 (1.52)	0.013
Safe to walk/jog in neighborhood	3.91 (1.21)	3.99 (1.19)	3.83 (1.26)	
Walkers and bikers in neighborhood	3.92 (1.18)	4.02 (1.08)	3.85 (1.18)	
Traffic in neighborhood	1.90 (1.23)	1.90 (1.10)	2.21 (1.23)	<0.0001
Crime in neighborhood	1.80 (1.11)	1.81 (1.09)	2.21 (1.23)	<0.0001
Peers playing in neighborhood	3.65 (1.35)	3.63 (1.34)	3.35 (1.33)	0.0009
Interesting things in neighborhood	3.19 (1.37)	3.10 (1.31)	3.00 (1.30)	0.029
Neighborhood streets are well lit	3.22 (1.34)	3.18 (1.31)	2.98 (1.31)	0.005
Body Mass Index (kg/m^2^)	22.41 (5.12)	23.93 (5.41)	25.99 (6.65)	<0.0001
Self-management strategies	26.43 (6.09)	26.79 (5.90)	27.88 (6.35)	0.0003
Self-efficacy	28.48 (6.47)	28.43 (6.27)	28.48 (6.74)	
Enjoyment of physical activity	28.97 (6.02)	28.53 (5.77)	28.38 (5.93)	
Perceived barriers	21.43 (5.95)	21.87 (6.09)	19.57 (5.61)	<0.0001
Outcome-expectancy (Belief)	36.62 (5.66)	36.15 (5.18)	35.53 (4.77)	0.0012
Outcome-expectancy (Importance)	158.68 (45.53)	148.37 (47.90)	150.89 (33.15)	0.0024
Social support	1.66 (1.11)	1.44 (1.11)	1.51 (1.07)	0.02
Friend support	5.50 (3.01)	4.87 (3.07)	4.15 (2.90)	<0.0001
Family support	10.50 (5.02)	8.84 (4.86)	6.29 (4.80)	<0.0001
Depressive symptoms score	15.51 (9.52)	15.34 (9.10)	14.64 (9.28)	
Distance to nearest park (miles)	1.20 (1.38)	1.02 (0.95)	0.70 (1.56)	<0.0001
Distance to nearest school (miles)	0.55 (0.54)	0.55 (0.53)	0.46 (0.54)	0.011
MVPA (minutes/day)	20.83 (10.70)	19.89 (11.58)	16.30 (14.97)	
Median (Q1, Q3)				
Number of parks within 1 mile	1 (0,1)	1 (0,1)	2 (1,5)	<0.0001

### Main findings

**Fig 1:**
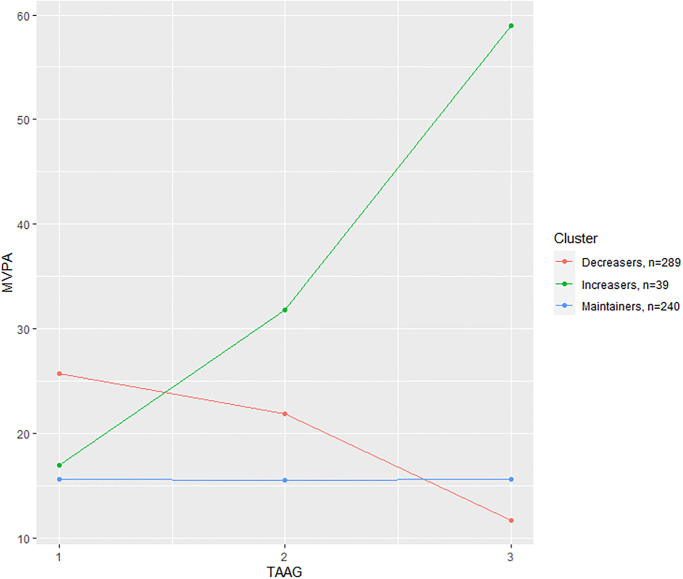
Mean MVPA trajectories in each cluster. Mean moderate-to-vigorous physical activity (MVPA) trajectories of the three clusters over the three TAAG waves at middle school, high school, and early adulthood, where the red line is for the Maintainer cluster, the green line is for the Decreaser cluster, and the blue line is for the Increaser cluster.

**Fig 2:**
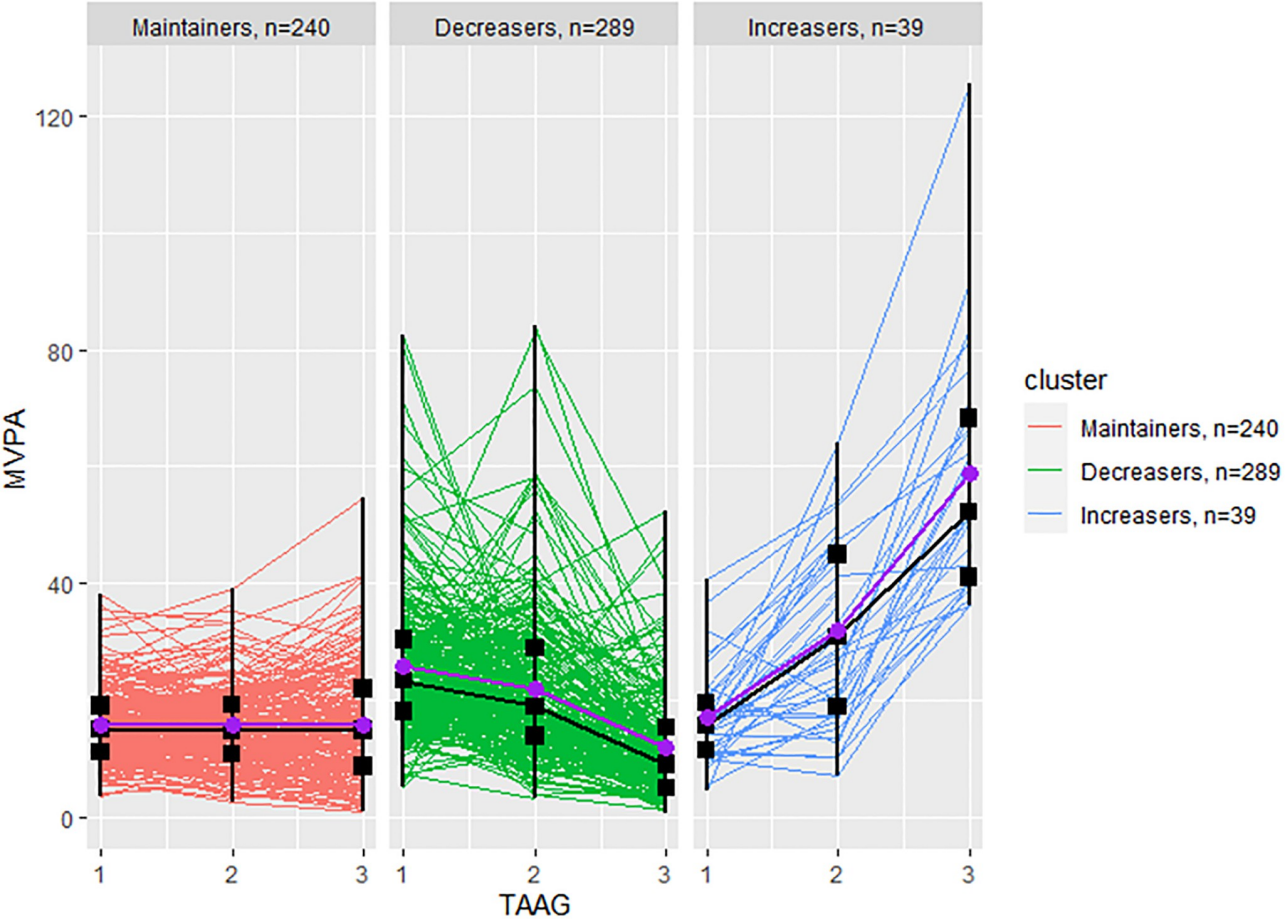
Individual MVPA trajectories in each cluster. Individual moderate-to-vigorous physical activity (MVPA) trajectories of the three clusters over the three TAAG waves at ages 14, 17, and 23, where the red lines are for the Maintainers, the green lines are for the Decreasers, and the blue lines are for the Increasers. The median and interquartile range per TAAG wave in each cluster are plotted over the trajectories in black, and the mean per TAAG wave in each cluster is plotted over the trajectories in purple.

There were 568 participants included in the analysis. Based on the BIC values, the optimal model identified three clusters of participants with similar physical activity patterns shown in Figs 1 and 2. The first cluster consisted with 240 participants with consistently low MVPA (Maintainers) over time (age 14: 15.59 ± 6.17 minutes/day, age 17: 15.56 ± 6.85 minutes/day, age 23: 15.56 ± 9.39 minutes/day); the second cluster is the largest with 289 participants who had daily MVPA relatively high at age 14, but decreased over time (Decreasers) (age 14: 25.74 ± 11.69 minutes/day, age 17: 21.82 ±12.45 minutes/day, age 23: 11.71 ± 9.27 minutes/day); the most rare group had only 39 participants with daily MVPA low at the young age, but increased over time (Increasers) (age 14: 16.93 ± 7.91 minutes/day, age 17: 31.83 ± 15.20 minutes/day, age 23: 58.95 ± 21.01 minutes/day). The clustering results are consistent with the previous finding [17], despite including more participants.

The variables selection and model estimation results for the three clusters are displayed in Table 2. As a comparison, the variable selection and model estimation results in Young et al. (2018) are also listed in Table 2 (column 1). For all three clusters (columns 2–4 in Table 2), the estimated intercepts represent the MVPA mean baseline level with all variables equal to zero. The variable *TAAG* (can be considered as a time/wave indicator) was selected in all clusters as an important indicator of the MVPA change over time. In the Maintainer group (column 2 in Table 2), the 240 girls had an intercept of 14.01 minutes of MVPA per day. The coefficient -1.10 for *TAAG* indicates a slight decrease in MVPA after each TAAG wave. The other two variables selected in addition to TAAG were *friend support* and the *number of parks within 1 mile of participants’ homes*. Both were positively associated with MVPA, which means that participants with higher friend support and a larger number of parks within 1 mile of their home were more likely to have higher MVPA levels in this cluster.

**Table 2:** Variable selection results in each cluster compared with the previous study.

	Young et al. (2018)	Maintainer	Decreaser	Increaser
		*n* = 240 (42.3%)	*n* = 289 (50.9%)	*n* = 39 (6.8%)
	(*N*_*β*_ = 8)	(*N*_*β*_ = 3)	(*N*_*β*_ = 7)	(*N*_*β*_ = 5)
Intercept	16.14	14.01	32.36	1.74
TAAG	-2.56	-1.10	-16.16	29.11
Places to walk near home	0.29			
Interesting things in neighborhood	0.35			
BMI	-0.16			
Self-management strategies	0.22		0.81	0.34
Friend Support	0.24	0.15		
Mother Education	0.86			
Number of Parks	0.71	0.39		
Traffic				-3.62
Distance to near park				2.36
Self-efficacy			0.37	
Outcome-expectancy (belief)			-0.42	
Outcome-expectancy (importance)			-0.019	
Social Support			-1.75	
Family Support			-0.27	
Distance to school				5.37

In the Decreaser group (column 3 in Table 2), the 289 girls started with a mean 32.36 minutes of daily MVPA, which was higher than the increaser group, but had a significant decrease of 16.16 minute/day in MVPA on average after each wave, indicating a large decrease over time. Six variables in addition to *TAAG* were included in the model. The results show that in this cluster, participants with higher *self-management strategies*, higher *self-efficacy*, *lower outcome expectancy* tend to have higher MVPA levels for the group of girls. We also found *social support* and *family support* was negatively associated with MVPA levels, which needs a further exploration.

In the Increaser group (column 4 in Table 2), although it only comprises 6.8% of the study sample, is the most interesting group. The 39 girls had an intercept of 1.74 minutes of MVPA per day, their MVPA increased greatly by 29.11 minute/day on average after each wave on average. Four variables in addition to *TAAG* were selected. The results show that within this cluster, participants with higher *self-management strategies*, less *traffic in their neighborhood*, longer *distance to the nearest park* and *school* were more likely to have higher MVPA levels.

### Comparison to previous findings

The difference in the clustering results compared with those in Young et al. (2018) is summarized in Table 3. Note that the goals in the paper of Young et al. (2018) differed from ours as their primary goal was to identify the factors associated with the longitudinal change in MVPA for the whole study sample and the the clustering was only applied to the outcome trajectories with no explanatory variables involved; while our goal is to group the girls with similar MVPA trajectory patterns and find the factors associated within different patterns. One can see from the diagonal entries that most of the participants were still clustered into the same groups. The major difference is that 94 participants who were clustered into the Maintainer group were clustered into the Decreaser group in our analysis. Many of these participants had decreasing MVPA over time. For example, one participant with MVPA over the TAAG time points was: 29.1 minutes/day, 12.6 minutes/day, 9.3 minutes/day was clustered into the Maintainer group in the previous analysis but was clustered into the Decreaser group in our analysis. Another example is a participant with MVPA trajectory such that 14.2 minutes/day, 15.8 minutes/day, 5.4 minutes/day was moved to the Decreaser group from the Maintainer group. Consequently, the mean MVPA level remained almost the same in our Maintainer group (age 14: 15.59 minutes/day, age 17: 15.56 minutes/day, age 23: 15.56 minutes/day) compared to the Maintainer group in Young et al. (2018) (age 14: 16.2 minutes/day, age 17: 13.9 minutes/day, age 23: 12.6 minutes/day).

**Table 3:** Comparison of the clustering results with the previous analysis.

		New model			
		Maintainer	Decreaser	Increaser	Total
Young et al, 2018	Maintainer	176	94	4	274
	Decreaser	20	103	0	123
	Increaser	5	4	22	31
	Total	201	201	26	428

The difference in the variable selection results compared with Young et al. (2018) is summarized in Table 2. Four out of eight variables selected in the previous study were selected in at least one cluster and the signs are mostly consistent. In addition to the variables selected in the previous study, the model recognized more cluster-specific variables that were not identified when included in the previous model. In the previous analysis, the coefficient of TAAG which was estimated as -2.56 indicates a small decrease of MVPA in average for the overall population. However, in our analysis, each cluster has a unique TAAG coefficient that characterizes the MVPA change over time more accurately; in the Maintainer group, two of the previously selected variables were included; in the Decreaser group, only self-management strategies from the previous analysis was included. In addition, our analysis identified five more variables that were not recognized in previously; in the Increaser group, again only self-management strategies from the previous analysis was included and three new variables were recognized.

## Discussion

Compared to the previous analysis, our results identified distinct clusters of MVPA trajectories as well as cluster-specific longitudinal predictors of MVPA within each cluster simultaneously. Three patterns of MVPA change over time are clearly revealed and different predictor variables are identified to be associated with the response in different clusters. Compared to the previous findings, variables being selected have overlap, e.g., the time indicator (TAAG) was selected in both studies. However, the coefficients of -2.56 in Young et al. (2018) showed a decreasing trend of MVPA in the overall sample, while the different coefficient values in our analysis better summarizes the trends as maintainers (TAAG = -1.10), decreasers (TAAG = -16.16), and increasers (TAAG = 29.11). Different variables were selected in the two studies due to the different research purposes and different analytic methods.

The results indicate that to improve MVPA among adolescent girls, the same strategies may have different effect on adolescent girls with different MVPA trajectories. However, self-management strategies was found to predict MVPA in both the decreaser and increaser groups. These results suggest that interventions that encourage the development of self-management strategies in early adolescence and then encouraging their use and continued development during later adolescence may be important to increase MVPA can lead to higher level physical activity irrespective of the overall change over time.

Other than self-management strategies, the other predictors for the increasers group are related to the home environment. Reporting less traffic and living farther away from schools and parks predicted greater MVPA. We do not know what types of neighborhoods the participants lived in over the three measurement periods. A systematic review of neighborhood features found that more walkable neighborhoods and good quality parks and playgrounds were associated with more MVPA [31].

Not just the nearness of parks, but their quality, availability of amenities, and freedom of crime are important factors that we were not able to assess. The clustering technique for variable selection based on the linear mixed-effects model has a great potential for longitudinal and multi-level data. It is an automatic procedure to group similar participants and identify the most important variables in each cluster and their cluster-specific association with the outcome trajectories. This procedure is completely data-driven, no screening or prior knowledge of the data is required. This one-step procedure was employed to analyze the TAAG data. Two-step procedures used in the previous analyses [17, 32] performed clustering and variable selection via two separate steps and the assumptions differ between the two steps: the clustering step assumes the sample was drawn from a mixture of populations, but the variable selection step assumes the sample was drawn from a homogeneous population. The one-step procedure closes the gap between the two assumptions and assumes the sample was drawn from a mixture of populations. Such assumption is more realistic for a large and diverse sample like TAAG. Other strengths of this procedure include the variable selection technique which allows us to include a comprehensive set of multilevel variables that associated with MVPA. The variable selection technique considers all variables simultaneously thus no prior knowledge about which variables to include is required. It reveals the crucial features related to the longitudinal outcome from a large pool of candidate features. Despite the high-dimensional input, only a subset of them are truly associated with the outcome. The variable selection technique is able to reduce the dimensionality by eliminating irrelevant variables from the model. The resulted model has meaningful interpretation and is useful for future studies.

The novel procedure also has limitations. The method might miss some variables in certain situations. For example, when a variable has a large between-cluster difference but a very small within-cluster variation, such a variable will be absorbed by the intercept of the linear mixed-effects model within the cluster. In the TAAG application, the variable mother’s education, which was selected in Young et al. (2018) and showed positive association with MVPA in the overall population, is significantly different between clusters in the three identified clusters such that the increaser group has the highest percentage of “college or more” level, as shown in the S1a Fig. However, this variable was omitted in our variable selection analysis because the variable has very small variation within clusters. In other words, within each cluster, the participants had very similar mother’s educational levels thus the effect of mother’s education on MVPA was absorbed and cannot be identified. Another situation is when the variable has a high collinearity with another variable (e.g., time). If the within-cluster variation becomes small after controlling for the effects of the correlated variable (e.g., time), it will not be identified by the method. In the TAAG example, BMI, which was selected and showed a negative association with MVPA in the overall population in Young et al. (2018), is highly correlated with time with a significant cluster difference between the resulted clusters. After the time indicator TAAG being included in the model, participants had very similar BMI values within clusters, i.e., BMI increased over time but at each time point the BMI values are very similar (small variation) within each cluster. The details can be seen in the S1b Fig. Thus, it will not be selected into the linear mixed effects model in each cluster. Mother’s education and BMI were identified in Young et al. 2018’s analysis because the linear mixed-effects model was applied to the whole sample by assuming a homogeneous population, the variation caused by them between clusters therefore was recognized. However, if a linear mixed-effects model was fitted for each cluster, the variation is no longer present and thus impossible to be identified. The same method can also be applied when researchers would like to demonstrate whether the effects selected in each cluster are significant. We could therefore argue from the other side that the method successfully grouped the participants with similar patterns into three clusters. It did such a nice job, and the participants are so similar within clusters and the variation becomes negligible to be identified. From this angle, this is actually not a limitation but more like an advantage. Nevertheless, for the purpose of clustering and understanding the cluster-specific association between response variable and predictors, omitting such variables does not deteriorate the clustering or model performance.

As one can see from the discussion above, post-selection inference in clustering methods is even more challenging than the inference problem for single models because one should consider not only the within-cluster but also between-cluster significance. In the paper of Yang and Wu [33], the authors proved that joint convergence rate of the fixed and random effects when both dimensions grow at an exponential rate of sample size within clusters (i.e., for a homogeneous population). Under the same setting, they also proved the sparsistency property. However, the construction of confidence intervals was not provided. As suggested by one reviewer, in each cluster we re-fitted the model using the selected variables to test the within-cluster significance. Some variables are not significant within clusters; however, they might be important variables to distinguish clusters. For example, the time indicator TAAG, which is insignificant in cluster 1 (maintainers, whose MVPA remained at a similarly low level at all three time points), but it is significant and has different effects in clusters 2 (decreasers) 3 (increasers). Our analysis successfully selected the TAAG variable in all three clusters despite its significance level.

## Supporting information

S1 Fig Repeated measures ANOVA analysis of selected variables by the clustering method. Plots of (a) counts in Mother’s Education and (b) mean BMI over time in the three clusters. The mean square error at each time point, within-cluster mean square error and between-cluster mean square error are provided below each plot and * indicates significant difference between groups.
(TIFF)

S1 Table Description of TAAG variables. Detailed description of variables used in the TAAG analysis.
(PDF)
