# Supplementary figures and images for: Clustering of longitudinal physical activity trajectories among young females with selection of associated factors
Source: PLoS One. 2022 May 12;17(5):e0268376. doi: 10.1371/journal.pone.0268376 (PMC9098033; doi:10.1371/journal.pone.0268376)

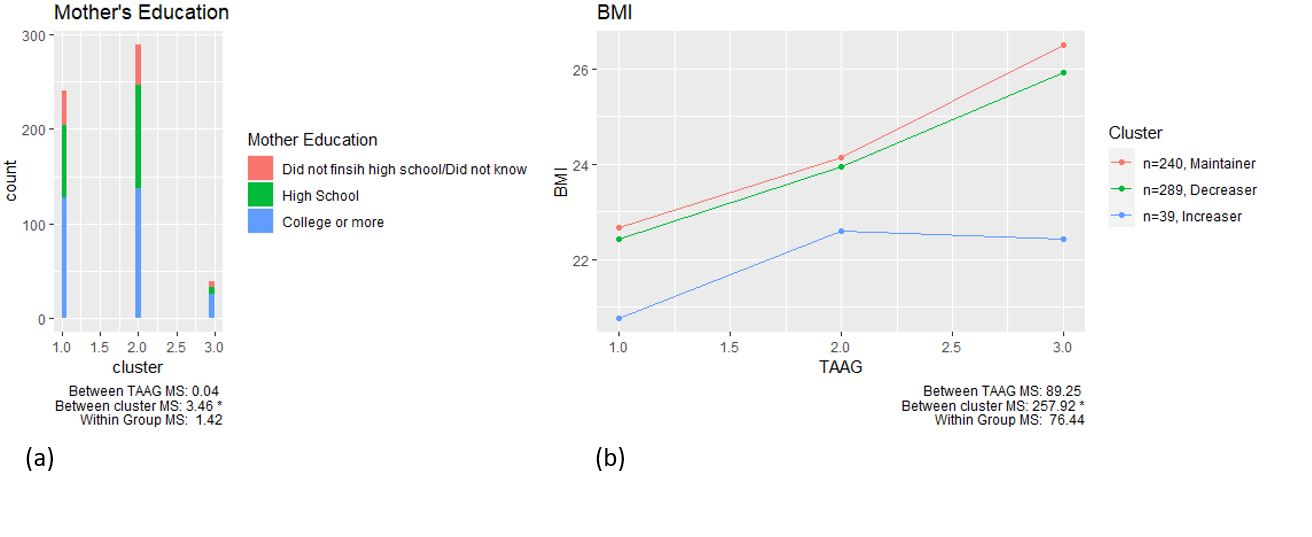

Supplement: S1 Fig — Plots of (a) counts in Mother’s Education and (b) mean BMI over time in the three clusters. The mean square error at each time point, within-cluster mean square error and between-cluster mean square error are provided below each plot and * indicates significant difference between groups. (TIFF) [file pone.0268376.s001.tiff]
