# Supplementary material for: Clustering of longitudinal physical activity trajectories among young females with selection of associated factors
Source: PLoS One. 2022 May 12;17(5):e0268376. doi: 10.1371/journal.pone.0268376 (PMC9098033; doi:10.1371/journal.pone.0268376)
Supplement: S1 Table — Detailed description of variables used in the TAAG analysis. (PDF) [file pone.0268376.s002.pdf]

Supplementary Materials

**Variable**	Measure	Value
**TAAG**	Time indicator when the data were collected	0 - Middle School (baseline)<br>1 - High School<br>2 - Early Adulthood
**Place near home**	There are many places I like to go within easy walking distance of my home	Choose one of the following:<br>1 - Never (The statement is never true).<br>2 - Rarely(The statement is rarely true).<br>3 - Sometimes (The statement is sometimes true).<br>4 - Often (The statement is often true).<br>5 - Very often (The statement is very often true).
**Sidewalk**	There are sidewalks on most of the streets in my neighborhood.	
**Trail**	There are bicycle or walking trails in my neighborhood	
**Safety**	It is safe to walk or jog in my neighborhood	
**Walker/Biker**	Walkers and bikers on the streets in my neighborhood can easily be seen by people in their homes	
**Traffic**	There is so much traffic that it makes it hard to walk in my neighborhood	
**Crime**	There is a lot of crime in my neighborhood.	
**Peers**	I often see other girls or boys playing outdoors in my neighborhood.	
**Sight**	There are many interesting things to look at while walking in my neighborhood.	
**Light**	My neighborhood streets are well lit at night	
**BMI**	A measure of body fat based on height and weight	(weight (in kg)/height (in m))^2
**Race: White**<br>**Race: Black**<br>**Race: Hispanic**		1-Yes<br>0-No
**Self management**	The computed sum of 8 questions about self-management stragety (e.g. I do things to make physical activity more enjoyable)	Range from 8 to 40 by choosing one of the following in each question<br>1- Never<br>2 -Rarely<br>3-Sometimes<br>4-Often<br>5-Very often
**Self efficacy**	The computed sum of 8 questions about self-efficacy (e.g. friend or someone else to do physical activity with me)	Range from 8 to 40 by choosing one of the following in each question<br>1-Disagree a lot<br>2-Disagree a little<br>3-Neither Agree nor Disagree<br>4-Agree a little<br>5-Agree a lot

**Enjoyment**	The computed sum of 7 questions about enjoyment of physical activity (e.g. When I am active I am bored)	Range from 7 to 35 by choosing one of the following in each question<br>1-Disagree a lot<br>2-Disagree a little<br>3-Neither Agree nor Disagree<br>4-Agree a little<br>5-Agree a lot
**Perceived barriers**	The computed sum of 10 questions about perceived barriers to physical activity (e.g. How often does bad wether keep you from being physically active?)	Range from 10 to 50 by choosing one of the following in each question<br>1- Never<br>2 -Rarely<br>3-Sometimes<br>4-Often<br>5-Very often
**Motivation**	The computed sum of 9 questions about the motivation of getting physically active (e.g. physically active to get in shape)	Range from 9 to 45 by choosing one of the following in each question<br>1-Disagree a lot<br>2-Disagree a little<br>3-Neither Agree nor Disagree<br>4-Agree a little<br>5-Agree a lot
**Importance**	The computed sum of another 9 questions about the importance of getting physically active (e.g. in shape is important)	Range from 9 to 45 by choosing one of the following in each question<br>1-Disagree a lot<br>2-Disagree a little<br>3-Neither Agree nor Disagree<br>4-Agree a little<br>5-Agree a lot
**Social support**	During a typical week, how often encourage friends play	0-Never<br>1-Once<br>2-Sometimes<br>3-Almost everyday<br>4-Everyday
**Friend support**	The computed sum of 3 questions about friend support for physical activity (e.g. during a typical week how often do friends encourage you to play)	Range from 0 to 12 by choosing one of the following in each question<br>0-Never<br>1-Once<br>2-Sometimes<br>3-Almost everyday<br>4-Everyday
**Family support**	The computed sum of 5 questions about family support for physical activity (e.g. during a typical week how often do household members encourage you to play)	Range from 0 to 20 by choosing one of the following in each question<br>0-Never<br>1-Once<br>2-Sometimes<br>3-Almost everyday<br>4-Everyday
**Depression**	The computed sum of 20 questions about depressive symptoms (e.g. how often have you felt poor appetite)	Range from 0 to 60 by choosing one of the following in each question<br>0-Rarely or none of the time<br>1-Some or a little of the time<br>2-A lot of the time<br>3-Most or all of the time
**Smoking**	Whether the participant smokes or not during the past 30 days	1-Yes; 0-No
**Father Education**	Father's highest education	0-Did not finish high school<br>1-Finished high school<br>2-Went to vocational school<br>3-Took some college<br>4-Graduated from college or university<br>5-Has professional training beyond a 4-year college degree<br>6-Don't know
**Mother Education**	Mother's highest education	

**Distance to school**	Distance from a residence to the nearest school in miles
**Distance to park**	Distance from a residence to the nearest park in miles
**Number of parks**	Number of parks within 1 mile of a residence

Table S1: **Description of TAAG Variables.** Detailed description of variables used in the TAAG analysis.
